# Skin Malignant Melanoma and Matrix Metalloproteinases: Promising Links to Efficient Therapies

**DOI:** 10.3390/ijms25147804

**Published:** 2024-07-17

**Authors:** Angela Madalina Lazar, Daniel Ovidiu Costea, Cristiana Gabriela Popp, Bogdan Mastalier

**Affiliations:** 1 Faculty of General Medicine, University of Medicine and Pharmacy “Carol Davila”, 050474 Bucharest, Romania; bogdanmastalier@yahoo.com; 2 General Surgery Clinic, Colentina Clinical Hospital, 020125 Bucharest, Romania; 3 Second Surgery Clinic, Constanta District Clinical Emergency Hospital, 900591 Constanța, Romania; 4 Department of Surgery, University of Medicine and Pharmacy “Ovidius”, 900470 Constanta, Romania; 5 Pathology Department, Colentina Clinical Hospital, 020125 Bucharest, Romania; brigaela@yahoo.com

**Keywords:** malignant melanoma, skin cancer, metastases, matrix metalloproteinases, prognostic, pathogenic mechanisms, risk factors, treatment

## Abstract

Skin malignant melanoma (MM) is one of the most frequent and aggressive neoplasia worldwide. Its associated high mortality rates are mostly due to its metastases, while diagnosis and treatment of MM in its early stages is of favorable prognostic. Even skin superficial MMs at incipient local stages can already present with lymph node invasion and distant metastases. Therefore, knowledge of the controllable risk factors and pathogenic mechanisms of MM development, spreading, and metastatic pattern, as well as early diagnosis, are essential to decrease the high mortality rates associated with cutaneous malignant melanoma. Genetic factors are incriminated, although lifetime-acquired genetic mutations appear to be even more frequently involved in the development of MM. Skin melanocytes divide only twice per year and have time to accumulate genetic mutations as a consequence of environmental aggressive factors, such as UV exposure. In the search for more promising therapies, matrix metalloproteinases have become of significant interest, such as MMP-1, MMP-2, MMP-9, and MMP-13, which have been linked to more aggressive forms of cancer and earlier metastases. Therefore, the development of specific synthetic inhibitors of MMP secretion or activity could represent a more promising and effective approach to the personalized treatment of MM patients.

## 1. Introduction

Malignant melanoma (MM) is currently considered the most aggressive and deadliest form of skin cancer, with a high tendency to locoregional invasion and widespread metastases. Approximately a third of the MM patients develop metastases that frequently constitute the cause of death [1,2,3,4,5,6,7,8]. It is the 15th among the most common cancers worldwide and ranked the third among skin cancers (3–5% of the skin cancers), after basal cell and squamous cell carcinoma, but its incidence has significantly increased in the last decades (over the last 30 years), with almost a doubling incidence for every decade [1,5]. However, its associated mortality rates (75% of the deaths caused by cutaneous cancer) have remained similar or decreased very little with time despite the advancement in modern therapies, such as chemotherapy, targeted therapy, and immunotherapy. It is estimated that the lifetime risk of developing MM is 1 in 39 for men and 1 in 58 for women, especially due to the increased sun exposure and migration of northern fair-skinned people to warmer regions [5]. Its worldwide incidence varies a lot with the region and the amount of sunlight, the highest incidence rates being for Australia, New Zealand, South Africa, and the United States, while in Europe, the most affected countries are Denmark, Sweden, and The Netherlands [9,10].

### The Aims of the Review

As MM continues to associate a poor prognosis, thorough knowledge of its biological behavior, clinical presentation, modalities of diagnosis, and treatment are essential.

**One of the main aims** of the current review was to synthesize the current understanding of malignant melanoma, starting with its etiopathogenesis (risk factors, melanocyte and melanin pigment characteristics and role in MM development, genetic mutations that trigger malignancy, the mechanisms by which UV radiation and other risk factors trigger genetic mutations and MM), continuing with MM histopathologic types, diagnostic methods, surgical treatment, tumor staging, and prognosis according to the tumor stage, as well as a synthesis on MM metastases. However, the current treatment does not prevent distant metastasizing and has a poor prognosis for the patients.

In such a context, **the other main aim** of the review was to present the current understanding of the role of matrix metalloproteinases in the development, aggressiveness, and metastasizing behavior of MM and the putative use of matrix metalloproteinases (MMPs) as therapy targets. After a brief description of background information on the main MMPs that intervene in MM development and progression, we continue with a synthesis of the most relevant MMPs’ inhibitors (MMPIs) that have been tested in clinical trials. The results and limitations of the use of existing MMPIs and future directions are an important chapter of the current review.

## 2. Current Understanding of Malignant Melanoma (MM)

### 2.1. MM Etiopathogenesis: Melanin Role in MM Development, Risk Factors, Hereditary and Acquired Genetic Mutations (Figure 1)

#### 2.1.1. Melanocytes and Melanin Role in MM Development

##### MM Risk Factors

MM develops from melanocytes located at the base of the epidermis, which are of neural crest origin. In fact, due to the embryologic origin from the neural crest, MM has the highest tendency to develop brain metastases of all the neoplasias [11]. The risk factors for the development of MM are of a genetic and environmental nature; the interaction between such categories of factors is of capital importance. Environmental factors refer to the UV radiation (both UV-A and UV-B) from sun exposure, as well as from tanning beds, while sunscreen lotions appear to have no preventive effect [4,10,12]. Apart from UV exposure, genetic mutations, and family history, other reported risk factors for MM development are fair skin, male sex, advanced age, and number of moles/nevi [1,2].

Physiologically, melanocytes divide rarely, only twice every year, and can therefore accumulate several genetic mutations. The melanocytes are the cells that are responsible for the production of melanin, a pigment that is transferred via cell fingerlike projections to the keratinocytes. Melanin plays an essential role as it absorbs UV radiation and, together with the physiological dead layer of superficial keratinocytes, protects the skin from the lesions caused by sun exposure. In response to sunlight, keratinocytes generate alfa-melanocytes-stimulating hormone (alfa-MSH) that binds to its receptor located on the melanocytes, the melanocortin 1 receptor, signaling for the synthesis of melanin. The melanin pigment that accumulates inside the keratinocytes acts as a protective shield for the nuclei against the potential DNA-damaging lesions of UV radiation [5]. Two forms of pigment can be produced: eumelanin, which is brown/black, associated with darker skin, playing a key protective role, as it absorbs UV radiation better; pheomelanin, which is a yellow/red pigment, associated with fair skin and light eyes, that is less protective against UV damage, with the generation of carcinogenic reactive oxidative species in response to UV exposure. Therefore, skin, hair, and eye coloration, as well as the risk of developing malignant melanoma following sun exposure, are determined by the melanocortin 1 receptor genetic polymorphisms that determine its level of activity. The stimulation of an entirely active MC1R determines the production of darker eumelanin in darker-skinned people, with a lower risk of cancer after sun exposure. Instead, the stimulation of a partially functioning MC1R, in the case of lighter skin people, signals for the production of pheomelanin, with an increased risk of mutations following UV radiation exposure [5].

**Figure 1:**
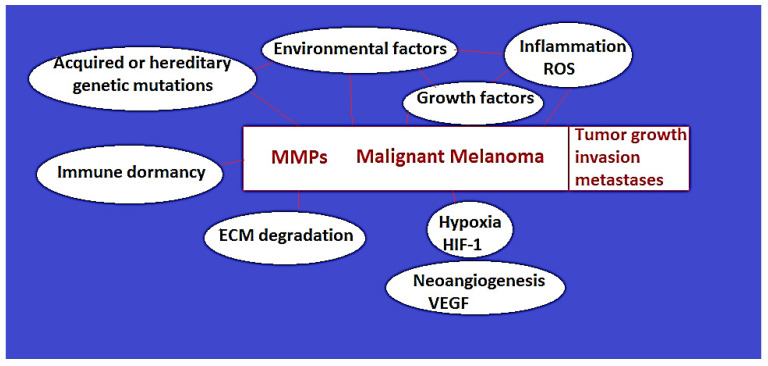
The role and interrelations of MMPs and other factors in the development, progression, and metastases of MM.

#### 2.1.2. Hereditary Genetic Factors

Some of the genetic factors that are responsible for the development of malignant melanoma are hereditary, with the inheritance of mutated genes, while others are lifetime acquired, although the profiles of the genetic mutations in the two cases are completely different. It is estimated that only a small percentage, 5–12%, of malignant melanoma cases are hereditary. For such cases, an individual that is diagnosed with malignant melanoma typically presents with many skin nevi and has a familial melanoma syndrome or a history of other familial genetic syndromes, such as xeroderma pigmentosum, mela-noma-astrocytoma syndrome, melanoma–pancreatic cancer syndrome, hereditary retinoblastoma, or familial atypical multiple mole syndrome [2,7]. Approximately 40% of familial melanoma cases are characterized by the mutation of the CDKN2A gene that determines defects in the p14 ARF and p16 INK4A proteins that are essential in controlling cell cycle arrest. However, such a genetic mutation can also be lifetime-acquired. In the case of the hereditary xeroderma pigmentosum, the mutations can occur in eight genes (XPA, XPB, XPD, XPE, XPF, XPG, XP-V) that normally control the DNA repair mechanisms via excision of the UV-damaged nucleotides. Such individuals with xeroderma pigmentosum lack the mechanisms of repair after UV-DNA damage and usually develop skin cancer early during their childhood [1,5].

#### 2.1.3. Lifetime-Acquired Genetic Mutations

However, the majority of the mutations that are associated with malignant melanoma are lifetime acquired. In such a scenario, malignant melanoma develops as a result of a significant accumulation of mutated genes when cell proliferation and death control/apoptosis mechanisms become severely disrupted [2,13]. Therefore, cutaneous malignant melanoma is significantly associated with elderly patients, more than 75 years old, who had time to accumulate sun exposure and genetic mutations [10]. The acquired mutations that occur in MM can affect the genes that are involved in the control of cell proliferation, such as BRAF, NRAS, and NF1 genes; in cell metabolism, PTEN and KIT; in cell cycle control, the cyclin-dependent kinase inhibitor gene-CDKN2A (around 18% of the genetic mutations) and cyclin-dependent kinase 4 gene (CDK4); cell replication, telomerase reverse transcriptase TERT promoter; cell resistance to apoptosis, TP53; and cell identity, ARID2. Of these, one of the most frequent is BRAF mutation, representing approximately 44% to 59% of the mutations, especially the V600 mutation, that leads to the activation of the MAP kinase signaling pathway involved in cell proliferation and survival [1,2,5,7,12,13,14]. However, such a mutation alone is not sufficient for malignant transformation, as it appears in the majority of benign nevi. The BRAF V600E mutation usually occurs after non-chronic, intermittent sun exposure, and it is associated with a superficial spreading type of melanoma that has a better prognostic than other types and is more promising in terms of targeted therapies [1,5].

#### 2.1.4. UV Radiation and Exposure to Other Risk Factors: Mechanisms of Triggering Genetic Mutations and MM

UV radiations and exposure to other risk factors and growth factors directly stimulate the MAP kinase pathway, signaling cell growth and proliferation. UV radiation also stimulates ERK, JNK, and p38 kinases that phosphorylate important transcription factors, with the activation of the genes that control cell growth, proliferation, differentiation, and death [5]. Usually, initially, a driver low-burden mutation occurs, such as either BRAF or NRAS mutation, that leads to melanocyte proliferation (melanocyte hyperplasia) or the occurrence of melanocytic nevi. If the genetic mutational burden increases, with TERT promoter mutations (TERT gene encoding telomerase reverse transcription), the benign melanocytic lesions transform into intermediate lesions and melanoma in situ. With the accumulation of more genetic mutations, such as mutations in the CDKN2A, TP 53, PTEN, or others, the lesion becomes invasive, that is, malignant melanoma [1]. Such transformations, with the final development of invasive cancer, occur as malignant melanoma is a highly immunogenic neoplasia, with immune editing and escape mechanisms with time, sometimes across many years. Initially, the normal immune system recognizes and eliminates the tumor cells (the elimination phase). Later, an equilibrium state is established, where the cancer does not progress, but it is not eliminated by the immune cells. At last, the escape phase ensues, when the immune defensive mechanisms are lost, and the tumor progresses [1,2,5,11,15].

### 2.2. Malignant Melanoma Types

Cutaneous malignant melanoma can occur in any localization of the body, with 20% of the cases in the head and neck region that associates a worse prognostic than that located in the extremities. Malignant melanoma can originate from a benign melanocytic nevus (in 20–40% of the cases), lentigo, or de novo within a normal area of unblemished skin without nevi [1,9]. It represents a heterogeneous disease with several histopathologic types that express different biological behaviors: superficial spreading; nodular; lentigo maligna; and acral lentiginous, amelanotic, desmoplastic, mucosal, and nevoid melanoma [16]. The most frequent type, accounting for approximately 70% of malignant melanoma cases, is the superficial spreading variant, which has radial growth and is better prognostic. Instead, the nodular type develops vertically and in-depth, with a higher risk of metastases, and is associated with a worse prognosis. Lentigo maligna type is rarer, developing slowly in the sun-exposed regions (face and head). The acral lentiginous malignant melanoma develops in the extremities, such as subungual space, hands, and soles, especially in darker skin patients. The desmoplastic variant is rare and occurs more frequently in the elderly. The most difficult and rare type to diagnose is the amelanotic type, which completely lacks pigmentation [6,7,10].

### 2.3. MM Diagnostic

The early diagnostic of cutaneous MM, ideally in situ, is of key importance, as the prognosis is directly linked to the tumor stage [1]. Clinical evaluation, screening of the lesions through dermoscopy and sequential digital dermoscopy, as well as other modern techniques, such as thermal imaging, tissue elastography, tumor cell genomic detection through non-invasive adhesive tapes, and identification of free blood circulating DNA from melanoma cells with BRAF mutations, are important tools that can be used for the early tumor diagnostic. Clinically, MM usually presents as an asymmetric lesion with an irregular tumor border, non-uniform coloration, and diameter of more than 6 mm that has an evolving behavior with changes in color, size, and shape [16]. However, even nowadays, the early diagnosis of malignant melanoma is difficult based on the histological analysis of the biopsy/resection specimen and immunohistochemistry [10]. For tumor diagnosis and staging, CT, PET-CT, MRI, and echography, as well as a modern minimally invasive liquid biopsy from blood or other fluid, with the detection of circulating tumor cells and DNA, can be used [12].

### 2.4. MM Tumor Stages, Treatment, and Prognostic

#### 2.4.1. MM Tumor Stages

According to the eighth malignant melanoma AJCC classification from 2018, there are five tumor stages, from 0 to IV, including a staging of the primary tumor (T), lymph nodes (N), and metastases (M) [16,17,18]. Stages 0 to IIC are considered early, as the tumor is confined to the skin, while no lymphatic or distant metastases are found. In the classification of the early stages, the primary tumor thickness (depth of the tumor invasion into the skin), that is, the Breslow thickness; Clark level of invasion; and the ulceration status are essential. Stage 0 represents in situ tumors; stages I and II are confined to the skin and subcutaneous fat. Stage III represents a locoregional spread of the cancer to the lymph vessels and lymph nodes. Stage IV is characterized by distant metastases [12,16,17].

#### 2.4.2. MM Treatment

In the early stages, the treatment is based on the complete surgical resection of the tumor with healthy surrounding tissue (negative margins). A 1–3 mm wide resection margin must be achieved in case of suspect lesions. However, in the case of established diagnostic of MM, the recommended extension of the resection margin depends on the tumor thickness. In the early tumor stages, a 1–2 cm margin is usually recommended as being enough; however, some authors consider that for a tumor thickness that is more than 2 mm, a wider resection margin of approximately 3 cm would be safer. A wider excision of 3 to 5 cm is not recommended for thin tumors under 2 mm. Other guidelines consider as follows: a 0.5 to 1 cm resection margin for in situ lesions; a 1 cm margin for MM that is less than 1 mm; a 1–2 cm margin for melanomas between 1 and 2 mm, and more than 2 cm margin for MMs that are greater than 2 mm [11,16,19]. Nonetheless, a complete tumor excision with safe margins is sometimes difficult to achieve in some body regions, such as the face and extremities [10]. Lymph node excision is not routinely performed. Sentinel lymph node biopsy is achieved only in the case of tumors that have a thickness of more than 0.8–1 mm or associate a metastasis risk of ≥5%. Radical lymphadenectomy (complete lymph node resection) is only performed if the sentinel lymph node or another clinically suspicious biopsied lymph node is positive [10,11,16]. The treatment of metastatic disease includes the surgical resection of metastases whenever it is possible and other therapies, such as radiotherapy, high-dose interferon, chemotherapy, targeted therapies, immune checkpoint inhibitors, and the offer of inclusion into clinical trials [11]. In advanced tumor stages, where surgical resection is impossible, the success of the pharmaceutical treatment depends on the tumor-associated genetic mutations, epigenetic modifications, and tumor microenvironment. As MAPK pathway genetic mutations are frequently associated with MM, targeted therapies, that is, drugs to inhibit MAPK and MEK, are used, such as a combination of Vemurafenib with Cobimetinib, Dabrafenib (RAF inhibitor) with Trametinib (MEK1/2 inhibitor), or Encorafenib with Binimetininb. Also, monoclonal antibodies against PD-1 (a protein that is expressed on the surface of tumor cells that inhibits T-cells), such as Pembrolizumab and Nivolumab, or against CTLA-4, have been developed [2,12,20].

#### 2.4.3. MM Patients’ Prognostic

The prognostic of the MM patients depends on several factors: the stage of the disease at the initial diagnostic, with the thickness and level of invasion (depth) index being of key importance; the localization of the MM, as the extremity localization associates better survival than those located in the trunk, head and neck regions; sex, which appears to be an independent prognostic factor, with men having a worse prognostic than females (that usually have extremity melanomas, with a better prognostic); white race; and age [1,2,9,10,16,19,21]. Fortunately, nowadays, due to the diagnostic progress, most cases are detected in earlier stages, with only a minority of patients presenting late, with distant metastases that are considered stage IV.

MM is curable in stage I, with complete surgical resection with negative margins, having an estimated 5-year overall survival of 95% for stage I and approximately 97.7% for the pT1 tumors [11,19,22].

Instead, the 5-year survival rate for stage IIC (ulcerated tumor with a more than 4 mm thickness) is reported to be approximately 53% [11]. In case of metastatic spread to the lymph nodes (stage III), the 5-year overall survival is 20–50% [19]. For stage III, the number of metastatic lymph nodes is of prognostic significance. For example, in stage III C, with four or more metastatic lymph nodes, any T, the reported 5-year overall survival rate was 28% [11]. For stage IV tumors, the level of LDH (lactate dehydrogenase) and the metastatic site are of prognostic significance. The highest one-year survival rates are for the distant skin and subcutaneous metastases (59%) (stage M1a) and for the lung metastases (57%) (M1b). Patients with other site metastases associate a worse prognostic, with a one-year survival rate of up to 41%. No matter the site of the distant metastases, if a patient has two high values of LDH (24 h apart), the case is classified as M1c [11]. The highest lethality is caused by lung metastases that lead to respiratory failure, followed by central nervous system metastases [16].

### 2.5. MM Metastases

Even for the early diagnosed cases, with complete surgical resection, there is a high metastasizing rate, with MM being characterized by unpredictable behavior. Therefore, even the incipient stage MM cases associate a poor prognostic sometimes with progressive disease, despite initially complete resection and a high mortality rate. Therefore, the current TNM classification appears non-ideal in patient risk classification and selection of the therapeutic regimen. In such a scenario, other prognostic essential factors should be identified and included in an improved classification for better stratification of the patients between low and high risk, where more aggressive therapeutic and follow-up regimens would be required [22].

Therefore, although a higher Breslow depth of invasion is associated with a higher metastasizing risk, even thin tumors can already present at the initial diagnosis with lymph nodes and/or distant metastases. It was reported that trunk, head, and neck tumors, uveal melanomas, and tumors with thickness between 0.75 and 1.5 mm were associated with the highest risk of metastases [11,22]. The metastases can occur anywhere, in various organs, with the most frequent sites being the following: distant skin and subcutaneous (10–60% of the cases); pulmonary (10–40% of the cases); brain (2–20% of the cases); liver (up to 20% of the cases); bone (4–17% of the cases); gastrointestinal (up to 8% of the cases); pleura; pancreas; kidney; thyroid (very rare); heart [8,20]. Although rare, metastases can occur even in the small intestine, with MM being one of the most frequent neoplasias that give metastases at such a site [11]. MM metastases can occur anytime in a lifetime, more frequently in the first 36 months from the initial diagnostic and resection, but were sometimes described after 5–10 years or even unexpectedly later, after 40 years from the initial treatment, highlighting the need for a prolonged, even lifetime follow-up of the patients [8,19,20]. Actually, the mean time interval from the diagnosis to the metastasis is 16 months for the lymph node metastases and 25 months for the distant metastases [11]. The occurrence of metastases many years after the initial diagnosis and treatment is possible due to the persistence of dormant tumor cell niches with various localizations after induction of immune system tolerance/suppression or even being protected by the immune cells [23]. The tumor stage at the initial diagnosis and its growth rate influence the time to the metastases [8,24]. Also, women who usually present with extremity MMs associate a longer time from the primary tumor diagnosis until the development of the distant metastases compared with men who develop trunk, head, and neck melanomas more frequently [21]. A longer time from tumor diagnosis to liver metastases and shorter for brain metastases was reported [8]. There are also cases of metastasis development in transplanted organs after 10 years from the recipient’s MM diagnostic [16]. Such reports highlight that even from the initial diagnostic in the early stages, there may already be meta-static clones in distant organs that remain dormant for long periods. At the metastatic site, equilibrium between the tumor process and the host defensive immune system occurs, preventing the occurrence of a macroscopic clinically evident metastasis. However, in case of immune vulnerability, the metastatic cells proliferate, and the tumor becomes clinically obvious. In the metastatic stage of disease, the prognostic is gloomy, with a median survival time of less than 12 months, around 6–8 months (from 2 to 12 months) [8,20,22]. The 5-year survival rate for the stages with lymph node or distant metastases is estimated at approximately 15–20%, and a 10-year survival rate of less than 10% [8,10,15]. The lymph node, distant skin, and subcutaneous metastases have the highest median survival time, while visceral metastases other than lung, liver, brain, and bone have a worse prognostic. Also, the number of distant metastases is of prognostic significance. The metastases can occur via the lymphatic route, via the hematogenous route, or both simultaneously [11,21]. There are three potential modes of tumor spread described: initial tumor- in transient/satellite metastases; primary tumor–regional lymph node, with further potential for distant metastases; initial tumor–direct distant metastases [21]. Therefore, knowledge of the metastatic mechanisms and an early diagnosis of the distant metastases are essential for an improved prognostic of MM patients [15].

## 3. Current Understanding of the Role of Matrix Metalloproteinases in MM (Figure 1)

### 3.1. Background Information on Matrix Metalloproteinases (MMPs)

Currently, an important percentage of patients are refractory to existing therapies or have poor results and still develop metastases even after timely complete surgical resection. Therefore, new, more efficient directions of research and treatment must be considered and developed. MM is associated with increased expression and activity levels of multiple MMPs [25,26,27,28]. Therefore, one of the actual research directions is directed toward the MMPs, with the analysis of their role in MM etiopathogenesis, as well as the design of MMPIs as novel, more promising therapies. Several studies, including ours, have highlighted the potential value of using MMPs’ expression for the prediction of MM metastatic behavior (occurrence and timing) (Table 1) [29]. Before presenting some of the key aspects that link MMPs to the development of MM, we briefly review some of the background information on MMPs.

Matrix metalloproteinases (MMPs), matrixins, or zinc- and calcium-dependent endopeptidases belong to the larger group of metalloproteinases that also includes ADAMs (a disintegrin and matrix metalloproteinases) and ADAMTS (ADAMs with thrombospondin motifs), which have key roles in the proteolysis of the extracellular matrix (ECM) and are able to degrade various ECM substrates. MMPs are physiologically produced by the epidermal cells (keratinocytes and fibroblasts) but also by immune (neutrophils, macrophages, dendritic cells) and tumor cells. Twenty-four human MMP genes and twenty-three MMPs have been discovered at present [30]. However, MMPs were numbered from 1 to 28 (three are non-human, and one MMP has been numbered twice) and classified according to their substrate as collagenases, gelatinases, matrilysins, stromelysins, membrane-type MMPs, metalloelastases, and other types. However, later, it was discovered that each MMP can degrade multiple substrates, while each substrate can be lysed by several MMPs [2,3,22,31,32]. Some MMPs are secreted extracellularly, and membrane-type MMPS play key roles in ECM remodeling, while the intracellular MMPs (inside the cytosol, organelle, or nucleus) currently have an insufficiently clarified role [30]. Some intracellular MMPs appear to cleave proteins such as TnI (troponin I), associated with cardiac pathology; βB1 crystallin, associated with the development of cataracts; and αβ-crystallin [30]. ECM proteolysis is essential in continuous physiologic tissue remodeling and cell–ECM interactions playing key roles in cell differentiation, migration, development, embryogenesis, angiogenesis, and wound healing [2]. However, ECM proteolysis by the MMPs is also at the basis of pathogenic processes, including inflammatory and autoimmune processes, such as arthritis, neoangiogenesis, and atherosclerosis; development of aortic aneurysms; varicose veins; cardio- and cerebrovascular diseases; degenerative nervous diseases, such as Alzheimer or Parkinson’s disease; glaucoma; cirrhosis; periodontal disease; lung fibrosis; and various types of cancer [33].

**Table 1:** Major features and roles of MMPs in malignant melanoma [2,22,34,35].

MMP Class (Type)	MMP Sub-Group	Sources	Physiologic Function	Role in MM
MMP-1 (interstitial collagenase)	Collagenases	Stromal cells (tumor-associated fibroblasts)	Fibrillar colagen degradation: collagen type I and III	MM invasiveness, vertical growth pattern, metastases; aggressiveness (non-regressive type)
MMP-2 (gelatinase A)	Gelatinases	Stromal cells, fibroblasts, endothelial cells, inflammatory cells, cardiomyocytes, benign melanocytic and MM cells	Collagen IV, as well as other non-fibrillar collagens (V, VII, X), gelatin, fibronectin, and type I collagen; contractile proteins, such as light chain myosin and troponin	MM radial growth pattern; tumor angiogenesis; aggressiveness and tendency to distant metastasizing; prognostic factor and predictor for vemurafenib resistance
MMP-3 (stromelysin-1)	Stromelysins	Stromal (fibroblasts) and tumor cells	Degrades collagen type I	Activates MMP-1, MMP-7, and MMP-13, associated with MM invasiveness and metastases
MMP-7 (matrilysin-1)-smallest MMP	Matrilysins	MM, tumor cells, stromal cells	Degrades elastin—degrades many ECM components, such as collagen IV, entactin, laminin, syndecan-1, cadherin, fibronectin, cartilage proteoglycans	Tumor invasiveness and metastases
MMP-9 (gelatinase B)	Gelatinases	Keratinocytes, fibroblasts, immune cells (neutrophils, macrophages), tumor cells	Gelatin, collagen type IV—component of the skin basement membrane, fibronectin, elastin, laminin, fibrillin; activates growth factors, including vascular endothelial growth factor, tumor necrosis factor-alpha, transforming growth factor beta, and fibroblast growth factor	MM radial growth pattern; promotes neoangiogenesis; putative anti-tumor effect in breast or colonic neoplasia; overexpression and activation of MMP-9 in the most aggressive forms of malignant melanoma; metastases
MMP-11 (stromelysin 3)	Stromelysins			Limited, contradictory knowledge in MM (MM progression versus regression)
MMP-12	Other types (Metalloelastase)	Fibroblasts, macrophages	Elastin	Tumor invasiveness, lymph node, and distant metastases
MMP-13 (collagenase-3)	Collagenases	Fibroblasts, especially tumor-associated cells	Collagen, gelatin, casein, as well as fibrinogen and growth factors	MM invasive vertical growth (metastatic)
MMP-23	Other types (a membrane-type MMP)	Membrane-type MMP, found in the endoplasmic reticulum and perinuclear membranes; Malignant melanoma cells	Cytokines, chemokines, blocks Kv1.3 on T-cells	Diminished immune anti-tumor cells response; resistance to adjuvant therapies

### 3.2. Physiologic Regulation of MMPs’ Level of Activity

#### MMPs Endogenous Inhibitors

**Figure 2:**
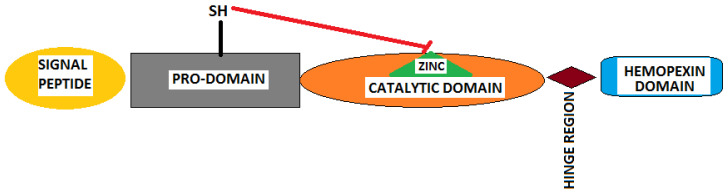
A schematic of the domains that can be found in all the MMPs in their pro-enzymatic state: a pro-domain that inhibits the zinc from the catalytic domain and that must be removed for enzymatic activation, a signal peptide (for secretion of MMPs; not present in membrane-type MMPs), a small hinge region, and the hemopexin domain that is responsible for substrate recognition and linkage.

MMPs are produced in inactive enzymatic forms of pro-enzymes that require cleavage for activation, usually achieved by other already active MMPs. They consist of an N-terminal domain; a signal peptide (pre-domain); a pro-domain (pro-peptide); a linker region; the catalytic domain, which is zinc (II)-linked (with a HEXXHXXGXXH zinc-binding motif); a hinge region; and a C-terminal hemopexin domain. The pro-domain, located towards the N-terminus, is an inhibitory sequence of 80 amino acids containing a cysteine switch motif that keeps the enzyme inactive because it is coordinatively linked to the catalytic zinc ion. It also contains a conserved PRCGXPD sequence across all MMPs. The C-terminus contains a conserved methionine turn. The hemopexin domain is responsible for the recognition and linking of the enzymatic substrates and inhibitors and interaction with other MMPs and TIMPs (tissue inhibitors of MMPs). The signal peptide or pre-domain, located at the N-terminus, is a signal for the MMP secretion; therefore, it is absent in membrane-type MMPs [2,30,33,34]. Figure 2 depicts the conserved domains across all MMPs in their pro-enzyme form (Figure 2). Some secreted MMPs, such as MMP-9 and MMP-2, are highly expressed in MM and contain three fibronectin-like repeats in the catalytic domain [2,30]. In MMP-2 and MMP-9, the catalytic zinc is coordinated by three histidines and glutamic acid. The most recently discovered MMP-23 has a less typical structure, with immunoglobulin-like and cysteine-rich domains and a transmembrane domain linked to the N-terminus [30,35].

MMPs’ level of activity is controlled at multiple levels, genetic and epigenetic: at the transcriptional level, activation of the enzymatic function, via factors that determine the inhibition of the enzyme, at MMP compartmentalization and complex formation [9,30].

The activation of the MMPs involves the removal of the inhibitory pro-domain (cyste-ine-switch model), either by cleavage or by allosteric conformational changes. In the catalytic/autocatalytic removal of the inhibitory enzymatic pro-domain, a sequence of approximately 80 amino acids must be cleaved and eliminated, allowing for the catalytic domain to become active and be able to act on substrates. Physiologically, the initial cleavage steps appear to be achieved by other MMPs (such as MMP-1, MMP-2, MMP-8, and MMP-9), as well as by plasmin, trypsin, urokinase, chymase, elastase [2,9,34,36,37,38]. Also, reactive oxygen species can trigger the activation of the enzyme [35]. Enzymatic activation can be a one-step process or several steps that take place for the full activation of the enzyme [30].

As already mentioned, there are also mechanisms of activation that do not require the removal of the MMP pro-domain by cleavage (the pro-domain remains intact). The linkage of certain molecules, including ECM degradation products, SIBLINGS (small in-tegrin-binding ligand N-linked glycoprotein) family members, human neutrophil lipo-cain, mercurial compounds, and molecules such as glutathione and peroxynitrite, determines an allosteric conformational change, and the catalytic unit of the enzyme is freed from the inhibitory influence of the prodomain; the result is in a partially or fully activated enzyme [30]. For cleavage, the substrate must interact with the catalytic domain. However, it must also interact with exosites (non-catalytic sites), which are regions outside the active site that play a role in the proper orientation of the substrate for enzymatic cleavage by the catalytic site [30].

Physiologically, there is a natural balance between the activity of the MMPs and their endogenous inhibitors, such as TIMPs (tissue inhibitors of matrix metalloproteinases), α2-macroglobulin, and α1-antiprotease. TIMPs intervene in a 1:1 stoichiometric ratio in inhibiting the activity of MMPs [6,9,36]. The MMPs’ inhibitors act by binding the zinc of the enzymatic catalytic domain. TIMPs interact with several MMPs, although they have inhibitory preferences. For example, TIMP-1 preferentially inhibits MMP-9, while it has no action on MMP-1 [2]. TIMPs are specific MMP inhibitors, but there are also endogenous non-specific MMP inhibitors, such as α2-macroglobulin, α1-antiprotease, tissue factor pathway inhibitors, GPI-anchored glycoproteins, membrane-bound β-amyloid precursor protein, and others [33].

### 3.3. Mechanisms behind the Increased Expression and Activity of Matrix Metalloproteinases in MM

Although there is a plenitude of studies on the implications of MMPs in MM, a smaller number of reports on the mechanisms that explain the increased expression and activity of MMPs in MM currently exist.

Physiologically, MMPs have a low level of activity that is in equilibrium with their endogenous inhibitors. However, MMP expression and activation are significantly increased in several cancer types, including MM, via multiple mechanisms [35]. In fact, MMPs appear to play a central role in the etiopathogenic mechanisms of MM, as depicted in Figure 1.

One of the most frequent genetic mutations associated with the development of MM, BRAF, leads to the constitutive hyperactivation of the MAP kinase, followed by the activation of the ERK transcription factor and the excessive expression of the MMPs, transforming factor-beta and osteopontin genes, responsible for tumor growth, invasion, and metastases [2].

Also, the development of tumor cells, triggered by genetic mutations, generates increased tissue pressure, hypoxia, inflammation, and reactive oxygen species that lead to the activation of signaling pathways, transcription factors, and genes responsible for stimulating the generation of MMPs (Figure 1) [35]. In particular, the dysregulation of the MAPK (Ras-Raf-MEK-ERK) and PI3K/AKT (PI3K/PTEN/AKT/mTOR) signaling pathways have been incriminated in the overexpression of MMPs, such as MMP-2 and MMP-9 [2] Also, alterations in RAC1B signaling pathway, with overexpression of FOXD1 (a neural crest associated gene), have been linked to MMPs up-regulation in MM. Many MM risk factors and genetic mutations are linked to dysregulated TGF (transforming growth factor)-signaling pathway and NF-kB activation, leading to increased osteopontin activation with up-regulated MMPs’ expression and activation in MM (such as MMP-9) [2].

Also, environmental risk factors, such as UV exposure or various chemicals, trigger inflammation, hypoxia, reactive oxygen species, and genetic mutations that lead to MMPs’ up-regulated expression levels (Figure 1). Reactive oxygen species (ROS) also increase the activation levels of the MMPs. ROS and other S-reactive agents, such as organo-mercurials, interact with the pro-domain cysteine, releasing the inhibition of the catalytic site [30]. Other factors that are known to up-regulate MMPs’ expression are inflammatory cytokines, growth factors, and hormones, including TNF-α, epidermal growth factor (EGF), TGF-β, basic fibroblast growth factor (bFGF), platelet-derived growth factor (PDGF), interleukin-1 β [34,39,40]. There is also an epigenetic up-regulation of MMPs. Once developed, melanoma cells secrete proteins and ncRNA-s (such as has-miR-155-5p, miRNA has-miR-296-3p, FOXC promoter upstream transcript (FOXCUT)) inside exosomes that are integrated by surrounding interstitial cells where they determine the inhibition of SOCS1 mRNA and activation of JAK-STAT signaling pathway. The results are an overexpression of the MMPs (such as MMP-9) and pro-angiogenetic factors (VEGF, FGF) genes [2]. Therefore, melanoma cells auto-stimulate their growth by increasing the activation of MMPs and pro-growth/pro-angiogenetic factors via the exploitation of the host’s normal surrounding cells.

At the same time, a dysregulation between MMPs’ expression and activity levels and their endogenous inhibitors, TIMPs, plays an already established role in the etiopathogenesis of MM [2,41]. Therefore, some MMPs’ inhibitors have been linked to cancer, as their expression is reduced or altered, especially in more aggressive invasive forms of neoplasia. Similar to MMPs, TIMPs’ expression is regulated by growth factors and cytokines. For example, TIMP-3 levels are usually reduced during tumor cell spread. In such a context, it was initially thought that the exploitation of endogenous inhibitors or similar synthetic compounds to inhibit MMPs could be used for cancer control. However, as some MMPs have anti-tumor roles, only some of the TIMPs also have anti-tumor and anti-angiogenesis effects. Instead, by inhibiting MMPs with anti-tumor effects, other TIMPs have an adverse, pro-tumor effect. For example, TIMP-1, which inhibits MMP-9, has been reported to present pro-tumor effects and is associated with cancer recurrence and poor prognosis. However, such an inhibitor can be exploited in atherosclerosis and aneurysms, where a higher MMP-9 activity was described [2,37,41,42,43].

## 4. MMPs’ Role in MM

### 4.1. MMPs’ Involvement in Tumor Development, Progression, and Metastasis

MMPs can intervene in the development, progression, and metastasis via multiple mechanisms. Several MMPs (Table 1) can intervene in ECM degradation by generating tissue pathways that are required for tumor development and spread. As the matrix components are proteolytically digested by the MMPs, with tumor cell–cell and cell–matrix detachment, free pathways inside the degraded extracellular matrix are created, where mobilized tumor cells start to spread, with consecutive tumor expansion. MMPs also act on other proteins, such as growth factors, cell receptors, chemokines, and inflammatory proteins, with the release of growth factors and hormones from the ECM that intervene in tumor growth and survival via immune escape mechanisms (Figure 1) [2,5,11,15,35,37,41]. The MMPs’ action on cytokines, as well as on immune receptors, such as the degradation of T-lymphocyte interleukin-2-receptor-α, is essential for tumor immune escape and gain of tumor tolerance that sustains cancer development and aggressiveness [35]. At the same time, MMPs act as tumor cell anti-apoptotic mechanisms that sustain tumor growth by cleavage of pro-apoptotic signals, such as the Fas ligand [34,41].

At the same time, ECM proteolysis achieved by the MMPs intervenes in the development of neovasculature that sustains the survival and promotes the growth of neoplasia at the primary, as well at metastatic sites [6,22,31,36,37,38,42,43,44,45]. Tumor angiogenesis appears to occur due to the hypoxic stimuli and vascular growth factors and chemokines that are released by the tumors and other stromal cells. Such factors are expressed in response to increased pressures and hypoxia within the tissues that are compressed by the tumors (Figure 1). MMPs intervene in the neoangiogenesis by releasing from the ECM and activating key pro-angiogenesis factors, such as vascular endothelial growth factor (VEGF), fibroblast growth factor (FGF), or transforming growth factor β (TGFβ) [2,35,41,46,47]. The pro-angiogenesis factors act as a signal and bind to endothelial cells, leading to blood vessels sprouting through the free territories created by the MMPs [2,42,43].

ECM degradation by the MMPs allows for tumor cell migration as a group. However, single tumor-cell spread can also occur. Neoplastic cells can acquire an epithelial-to-mesenchymal transition, with amoeboid-like movement, that is possible due to the cleavage of E-cadherin by multiple MMPs, such as MMP-9, MMP-10, or MMP-15 [41].

Following the proteolysis of the ECM as well as of the vascular basement membrane, the malignant melanoma cells enter the lymphatic and bloodstream. Some of the tumor cells are filtered and remain in the lymph nodes with the development of lymph node metastases, while the blood-entered cells are able to generate distant metastases anywhere in the body [22,37,42,48].

### 4.2. Characteristics of the Matrix Metalloproteinases That Play Roles in Malignant Melanoma Development and Progression

Several studies, including ours, have reported a particular significance for the expression levels of MMP-1, 2, 3, 9, 14, and 15 in MM [6,29,39,44,48] (Table 1). An increased level of activity, higher aggressiveness, and a potential predictive role for the occurrence of distant metastases were seen, especially for MMPS-2 and 9 [44]. However, later, the results have shown that only some MMPs hold a pro-tumoral function, while others may be protective against the development of cancer, including MM [2]. Table 1 lists some of the key features of the MMPs known to play roles in MM development, spread, and metastasizing, including their class, sub-group, source, physiologic function, and role in MM.

**MMP-1** (Table 1) is an interstitial collagenase, along with MMP-8 and MMP-13, that can degrade several types of fibrillar collagen. Collagenases are the predominant type of extracellular matrix endopeptidases, while MMP-1 is the most common. As collagen types I and III represent an essential part of the extracellular matrix, collagenases such as MMP-1 express a major role in its degradation and are also essential for tumor progression [2,3,22,36,45]. Therefore, besides its function in physiological processes, MMP-1 can also intervene in pathologic processes linked to the development and spreading of tumors, including MM. Increased MMP-1 expression was found to be associated with more aggressive MMs that have no spontaneous regression, contrasting with some melanoma types that exhibit such a feature. Instead, its expression is less pronounced in microinvasive and in situ MMs. The knockdown of MMP-1 in melanoma mouse cell lines was reported to decrease the tumor’s capacity to metastasize [22]. MMP-1 also intervenes in increasing the protease-activator receptor-1 (PAR-1) gene expression. That is, MMP-1 acts in the progression of the MM through the cleavage of the dermal collagen, but it also promotes the development of tumor neovasculature by stimulating the NF-kB signaling pathway, the expression, and activation of several vascular growth factors, such as vascular endothelial growth factor (VEGF), activation of the VEGF pathway, endothelial cells proliferation, and by increasing the level of expression of PAR-1 [3,42,43]. As increased PAR-1 expression promotes tumor vascularization, it is associated with MM depth of invasion [2,3]. Also, MMP-1 accumulated intracellularly during the cell cycle mitotic phase appears to protect from apoptosis, therefore promoting tumor cell survival, growth, and chemoresistance [38]. By degrading the extracellular matrix collagen, MMP-1 also has an important role in photoaging and skin degradation (as a result of UV damage) [3,36]. It also holds a role in HIV-associated neurotoxicity as it cleaves the Tat protein [2].

**MMP-2 (gelatinase A)** (Table 1) is an endopeptidase that degrades previously cleaved ECM components (by collagenases), such as collagen IV from the basement membrane, as well as other non-fibrillar collagens (V, VII, X); gelatin; fibronectin; and type I, II and III collagen, enabling tumor cell progression. It can also cleave contractile proteins, such as light chain myosin and troponin I [3,36]. It is mainly generated by the stromal cells, fibroblasts, endothelial cells, inflammatory cells (dendritic, macrophages, mast cells), and cardiomyocytes, but also by the benign melanocytic and MM cells in variable quantities [22,35,36]. It is generated as pro-MMP2 and activated by other MMPs and enzymes, such as MMP-14 [3]. It is believed that the level of MM aggressiveness and tendency of distant metastasizing is in direct relationship to its expression in melanoma cells, and it is proposed as a prognostic factor and as a predictor for vemurafenib resistance [22,29,49]. Its level of activity is decreased by angiotensin II and hypoxic stimuli, while collagen, inflammatory stimuli (cytokines, reactive oxygen species), and growth factors can increase its level of activity [3,36].

**MMP-9 (gelatinase B)** (Table 1) is much similar to MMP-2. MMP-9 is found in keratinocytes, fibroblasts, hematopoietic, immune cells (neutrophils, macrophages, lymphocytes), and tumor cells. It is secreted extracellularly into the matrix as a pro-enzyme of 92 kDa. Other already activated MMPs, such as MMP-2 and MMP-3, cleave the pro-enzyme into the active form of 84 kDa MMP-9. MMP-9 digests several components of the extracellular matrix, such as gelatin (that is denatured collagen), collagen type IV components of the skin basement membrane, fibronectin, elastin, laminin, fibrillin, and others. It also activates growth factors, including vascular endothelial growth factor, tumor necrosis factor α, transforming growth factor β, and fibroblast growth factor. It plays a role in photoaging and skin wrinkling, but it is also linked to tumor growth, invasion, and metastases, as it degrades the ECM and promotes neoangiogenesis [3,6,35,36]. The expression of MMP-9 is associated with a radial growth pattern of the MM [3]. However, in certain cancers, such as breast or colonic neoplasia, an anti-tumor effect was also described. The proteolysis of collagen IV by the MMP-9 releases tumstatin that inhibits the endothelial cells and, therefore, tumor angiogenesis [22]. Instead, in the most aggressive forms of MM, there is an overexpression and activation of MMP-9 and MMP-2. MAP kinase signaling pathway, frequently associated with BRAF mutations (with the hyperactivation of Ras-Raf-MEK-ERK, as well as the over-activation of the PI3K-PTEN-AKT-mTOR pathway or RAC1B-FOXD1 pathway), leads to the over-expression of several genes involved in tumor uncontrolled growth, proliferation, and spread, such as MMP-9, as well as MMP-2 genes. Also, osteopontin, a 300 amino acid protein normally produced from bone and other tissues (epithelia, mucosae, kidney) and overexpressed in tumors, binds to the cell integrin αvβ3, activating NF-kB and MAPK pathways, with the generation of MMP-9, resulting in tumor cell growth, proliferation, survival (anti-apoptotic), and metastases [2,11,49]. In such a context, an attempt was made to target and modulate the expression and activity of MMP-9 using specific microRNAs, such as miR-155-5p [2]. Some authors have reported that MMP-9 appears to be required for the development of the metastasis, while MMP-2 is for the progression of the primary tumor, particularly for the radial and not vertical growth [44]. Some studies, including ours, have found a correlation between MMP-9 tissue expression and serum circulating level and MM aggressiveness, which is considered a predictor for MM progression and development of metastases [29,48,50,51].

**MMP-3 or stromelysin-1** (Table 1), which degrades collagen type I, is known to activate MMP-1, MMP-7, and MMP-13 (Table 1), which is associated with MM invasiveness and metastases [3].

**MMP-7 or matrilysin**, involved in the degradation of ECM elastin, is produced by MM cells, secreted and activated extracellularly, and stimulates tumor growth and metastasizing [3].

**MMP-11 (stromelysin 3)** (Table 1) belongs to the stromelysin group of MMPs, along with MMP-3 and MMP-10. There is still limited knowledge on the roles of MMP-11 in malignant melanoma, with contradictory results. Some authors report a link between MMP-11 expression and tumor progression, while others have found that it may play a role in the regression of malignant melanoma [2,22,29,52].

**MMP-12** (Table 1), a metalloelastase that functions in the degradation of ECM elastin, as well as other ECM components (collagen IV, laminin, fibronectin, heparin, chondroitin sulfates, fibrillin-1 and others), is associated with solar elastosis after acute UV exposure, as well as with MM invasiveness and metastases (lymph node and distant metastases), which is regarded as a putative prognostic predictor [3,34].

**MMP-13 (collagenase-3)** (Table 1) can degrade several components of the ECM besides collagen, including gelatin, casein, fibrinogen, and growth factors. Its increased activity in atherosclerosis, with colagenolysis, is associated with unstable atherosclerotic plaques [3,36]. However, its increased level appears to be correlated to the mitotic index of the tumor cells as well, including MM [29,48]. It is associated with an invasive vertical growth (metastatic) pattern of MM [3].

**MMP-23 (a membrane-type MMP)** (Table 1) is found in the endoplasmic reticulum and perinuclear membranes, requiring a single cleavage for activation and secretion into the extracellular space. It can alter T cell functions by cleavage of cytokines and chemokines and blockage of Kv1.3 on T cells, with diminished immune anti-tumor response. Its high level of expression appears to be associated with a low response to adjuvant therapies, and MMP-23 inhibition could be an important therapeutic target [35,49].

## 5. MMPs as Therapeutic Targets: Knowledge of MMPs’ Inhibitors (MMPIs) and Their Experimental and Clinical Use in MM Treatment

The logic behind the use of MMPIs for patient treatment is based on the fact that the expression and activity of many MMPs’ are up-regulated in MM.

There are endogenous and exogenous MMPIs that can be exploited in MM treatment. The exogenous inhibitors are usually synthetic, although natural products have been proposed and tested as well in experimental and clinical practice [35].

### 5.1. Synthetic Inhibitors: Results in Preclinical and Clinical Trials and Limitations of Use

Some synthetic inhibitors are non-selective, as they bind to the zinc of the catalytic domain of the MMPs (zinc-binding inhibitors), which are hydroxamate-based compounds included in the first generation of developed inhibitors. Other inhibitors are more selective, with a narrower spectrum of MMP inhibition, such as catalytic non-zinc binding inhibitors (second generation of inhibitors, non-hydroxamate-based compounds), allosteric and exosite inhibitors, and antibodies against a specific MMP [2,33] (Table 2). Some inhibitors are peptidomimetic, while others are non-peptidic.

The synthesis of MMPIs began in 1900, with the first inhibitors being similar to collagen, the substrate recognized by the enzymatic catalytic site.

The first generation of synthetic MMPIs was hydroxamate-based, including Batimastat, Marimastat (BB-2516), Cipemastat (Ro 32-3555), and MMI-166. **Batimastat**, one of the first and most-tested MMPIs, was hydroxamate-based and peptidomimetic with a collagen-like backbone but had no selectivity, and it was able to inhibit all the MMPs. Also, because of its water insolubility and low oral bioavailability, it had to be injected into the peritoneal pleural space or into the mesenteric vein to target the liver. In several phase I studies, it was efficacious, inhibiting tumor growth and neoangiogenesis, as well as angiogenesis in liver metastases of malignant melanoma [34,41,53,54,55,56]. However, it also associated important toxicity with significant side effects, such as peritonitis, pain, nausea, pyrexia, alteration of liver function, cough, and musculoskeletal syndrome. The important toxicity and water insolubility, as well as the development of marimastat, which had oral bioavailability, led to the cessation of the clinical trials in phase III [34,41,53,54,55,57].

**Marimastat**, another peptidomimetic inhibitor, was similar to batimastat, with a hydroxamate group that bound and inhibited the catalytic zinc ion of several MMPs. It was an improved inhibitor when compared to batimastat, as it possessed oral bioavailability and was tested in phase II and III clinical trials for several types of tumors (gastric, pancreatic, colorectal, lung, breast, and prostate cancer). Although better tolerated than batimastat, further trials were not pursued, as no significant survival improvement was seen. Instead, it caused severe musculoskeletal syndrome, characterized by pain, inflammation, joint stiffness, muscle necrosis, gastrointestinal ulcers, and fatigue [41,54,55]. Musculoskeletal syndrome occurred because marimastat was an unselective inhibitor for MMPs, as well as for ADAM sheddases, normally responsible for TNF-α cleavage. Ensuing ADAM inhibition, there is an increased TNF-α level and, therefore, more inflammation. Also, excessive collagen deposits in the ECM and fibrosis occur in the absence of normal ECM degradation by MMPs, such as MMP-1, which physiologically degrades interstitial type I collagen [41,54,55].

Similar results to the use of first-generation inhibitors were also reported with the next generation of more selective and non-peptidomimetic MMPIs, which included Prinomastat, Tanomastat, and MMI 270 B. **Prinomastat**, a hydroxamate-based zinc chelator and **Tanomastat**, a thiol-based zinc-chelator, were more selective, inhibiting only MMP-2, MMP-3, MMP-9, MMP-13, and MMP-14, respectively, MMP-2, MMP-3, MMP-7 and MMP-9. However, such more selective inhibitors that have been associated less frequently with musculoskeletal syndrome still caused severe side effects such as joint inflammation with swelling and pain, bone marrow suppression (with anemia, thrombocytopenia), gastrointestinal disturbance, and venous thromboembolism (when associated to chemotherapy). Such severe side effects, as well as the lack of efficacy (no significant decrease in tumor growth), led to their cancellation in phase III clinical trials [2,41,54,57]. 

**Other second-generation inhibitors** were sulfonamide–hydroxamic acid-, phosphamides hydroxamic acid-, carboxylate-, thiolates-, phosphorus-, aminomethyl benzimidazole-, or nitrogen-based compounds. Such compounds are more selective MMPIs and are associated with lower toxicity and side effects [33].

**BAY12-9566**, a non-peptidic MMP inhibitor, biphenyl-based, which inhibits MMP-2, MMP-3, and MMP-9, has been tested in murine melanoma, but the results have led to the interruption of the clinical trials [55].

Other MMPIs were designed as **chemically modified antibiotics**, that is, modified doxycycline, tetracycline (Metastat, COL-3), and Minocycline, lacking antibiotic activity and inhibiting MMPs by zinc and calcium chelation [34,58]. Although not efficacious in MM or other tumors, doxycycline, which selectively inhibits MMP-2 and MMP-9, was proven to be clinically useful and approved for periodontitis prevention [34,55].

**Bisphosphonates**, such as letrozole, can inhibit MMP-2 and MMP-9, potentially being used in clinical practice to prevent metastases [34].

**Mechanism-based catalytic inhibitors** act by zinc coordination and triggering a conformational change in the catalytic domain that prevents the detachment of the inhibitor from the enzyme. The result is a stable enzymatic inhibition, achieved with a low inhibitor level, decreasing the risk of toxicity. For example, the use of a Thiirane-based ND-322 inhibitor resulted in favorable results in MM, with a decrease in tumor growth and delay of metastases [33,59].

In the search for more selective inhibitors, **catalytic non-zinc binding inhibitors** (such as PNU-141803 or PNU-142372) and **allosteric or exosite inhibitors** were also designed and tested. The allosteric inhibitors bind to the hemopexin domain of MMPs and trigger a conformational change that is less likely to bind substrates. Such allosteric inhibitors were already developed to inhibit MMP-2 and MMP-9, which are key enzymes in MM etiopathogenesis [33,57].

Other tested inhibitors are antibodies against MMPs, such as **antibodies** against membrane-bound MT1-MMP (MT1-MMP) and **recombinant tissue inhibitors of metalloproteinases**. For example, MT-1–MMP antibody LEM-2/15 led to a smaller number of lung tumors and decreased the dimension of the metastases in a mouse melanoma cell metastatic model [54]. **Monoclonal antibodies** against MMP-9 (andecaliximab, AB0041, AB0046, GS-5745) and against MMP-1, MMP-2, MMP-3, MMP-14, are currently under clinical studies. Of these, monoclonal antibodies against MMP-14 appeared to be efficient in MM preclinical studies [41,54]. Monoclonal antibodies have the advantage of being highly selective. They inhibit a specific MMP but can block only a specific function of an MMP or MMP activation by a specific molecule [33].

Other more promising molecules, with ongoing trials, are represented by **RNA interference inhibitors** of MMPs, with favorable preliminary results in MM studies, with prevention of developing metastases [46,60,61].

An insufficiently exploited therapeutic direction is the use of **anti-melanoma vaccines**. For example, syngeneic MMP-9 peptides were used to immunize mice against MMP-9, and the authors reported favorable positive results [25].

### 5.2. Natural Inhibitors

MMPs’ synthetic inhibitors frequently lack inhibitory specificity, with frequent inhibition of enzymes that are physiologically important for body homeostasis. The significant side effects associated with their use, as well as disappointing results in clinical trials, have led to the consideration of natural products as MMPIs in anti-tumor treatment, including MM. There are several natural MMPIs that are promising but still under research, such as shark cartilage; the soy isoflavonoid genistein; green tea extracts; monoterpene α-pinene from essential oils; selenium; retinoic acid; and nutrient mixtures containing lysine, proline, copper, manganese, N-acetyl cysteine, and vitamin C [34,39,62]. Also, some natural extracts from medicinal plants, such as caffeates and flavonoids, or marine algae (Neovastat, Ageladine A, Dieckol) can act as non-zinc-binding catalytic inhibitors. Their advantage is represented by low toxicity and cost; however, they require a high dosage to be effective, which is difficult to achieve [33].

### 5.3. Current Understanding of MMPIs’ Efficacy in Cancers, Including MM

Although initially, exogenous MMPIs appeared to have potentially important prognostic and therapeutic value, the clinical trials have not yielded favorable results, while other studies presented conflictive or inconclusive results. Instead, they have associated severe toxicity with important adverse effects, such as musculoskeletal syndrome (with important muscle and skeletal inflammation and pain), febrile neutropenia, and hypersensitivity reactions that led to the cessation of the clinical trials. Currently, the most promising MMPIs, with ongoing clinical trials, appear to be monoclonal antibodies against specific MMPs, interference RNA, and recombinant TIMPs.

### 5.4. Possible Explanations for the Disappointing Efficacy of MMPIs in MM

Many of the synthetic MMPIs lacked sufficient selectivity, inhibiting multiple MMPs, as well as other enzymes or molecules important for physiological processes (off-target interactions). Many have also associated dose-limiting toxicity [54,57,63]. Even if MMPIs are sufficiently selective, the inhibition of a pro-tumor MMP in MM can devoid the body of its physiologic action in other tissues, leading to unacceptable side effects. Also, the existing synthetic exogenous MMPIs have been clinically tested only in advanced tumor stages until now. However, in preclinical experiments, they were efficacious in limiting tumor growth and spread when delivered in the early stages or before tumor cell injection in laboratory animals. The preclinical studies did not reproduce the model of an advanced tumor stage. At the same time, when delivered to the tested animals before the tumor cell injection or in early stages, none of the tested inhibitors were able to prevent the occurrence of the tumor or of metastases, only diminished tumor growth. Such a finding generates important questions about our understanding of the MMPs’ roles in various tissues and microenvironmental conditions [33,54].

Another mechanism that could explain the lack of anticipated therapeutic efficacy with MMPIs is the variability of the MMPs’ action, with different putative results depending on the tested tissue, histopathologic type of cancer, tumor stage, and micro-environmental conditions [54].

Also, currently, there is a lack of objective, measurable, reliable, and easy-to-use indicators of MMPI effects in experimental studies that hinder their testing in clinical studies [33].

## 6. Future Directions

As the already tested MMPIs led to disappointing results in clinical trials, we can suspect that there are still missing pieces in our understanding of the MMPs’ and TIMPs’ role in MM.

Therefore, more research is required to understand all the mechanisms that regulate the expression and activity of MMPs and their natural inhibitors in MM. Such an advance in our understanding of the MMPs’ role in MM is essential for the designing and testing of improved targeted therapies, as many patients are refractory at current treatments and still associate poor prognostic.

The development of engineered endogenous-like inhibitors such as TIMPs with specificity for a certain MMP is one promising direction towards more efficient, targeted therapies. Also, testing monoclonal antibodies against specific MMPs, which proved to be linked to MM etiopathogenesis, is another direction that deserves significant attention in the future. Also, more research is required for the clinical exploitation of natural exogenous MMPIs, which would have the important advantage of non-toxicity and low production cost. Other promising therapies, with preliminary favorable results, are interference-RNA and even vaccines. To achieve such steps, new, innovative technologies for the design and delivery of specific small molecule inhibitors will be required.

## 7. Conclusions

As malignant melanoma is one of the deadliest skin cancers worldwide, with increasing incidence, knowledge of the risk factors and key pathogenic mechanisms that are at the basis of tumor development and spreading is of utmost importance. Lately, as several matrix metalloproteinases have been highlighted to play significant roles in malignant melanoma progression and metastatic behavior, new biomarkers of predictive value and promising therapeutic targets can be anticipated. As such, several studies have already found a significant association between MMP-1, MMP-2, MMP-9, and MMP-13 expression and tumor development, as well as aggressiveness and fast metastatic pattern. Therefore, synthetic inhibitors of MMP secretion or activity could be extremely promising for a better and individualized treatment of malignant melanoma patients. However, the clinical trials testing several synthetic MMPIs have not yielded the desired results, as such compounds also inhibit/alter other molecules essential for body homeostasis and are associated with significant side effects. Therefore, in the future, new compounds with increased selectivity, low toxicity, and good oral bioavailability should be designed. Of these, monoclonal antibodies, interference-RNA, recombinant TIMPs, and natural MMPIs appear promising for MM treatment.

## Figures and Tables

**Table 2:** Synthetic inhibitors of MMPs: types and mechanism of action.

**Synthetic Inhibitors**
**Catalytic inhibitors** Zinc-binding inhibitors, non-selective: - Hydroxamic-based compounds, non-selective; - Sulfonamide–hydroxamic acid-, phosphamides hydroxamic acid-, carboxylate-, thiolates-, phosphorus-, aminomethyl benzimidazole-, or nitrogen-based compounds that are more selective. Catalytic non-zinc binding inhibitors Mechanism-based inhibitors
**Allosteric and exosite inhibitors**
**Monoclonal antibodies against a specific MMP**
**Interference RNAs**
**Recombinant TIMPs**

## Data Availability

Data/further information are available by emailing the corresponding authors.

## References

[1] Eddy K., Shah R., Chen S. (2021). Decoding melanoma development and progression: Identification of therapeutic vulnerabilities. Front. Oncol..

[2] Napoli S., Scuderi C., Gattuso G., Bella V.D., Candido S., Basile M.S., Libra M., Falzone L. (2020). Functional roles of matrix metalloproteinases and their inhibitors in melanoma. Cells.

[3] Pittayapruek P., Meephansan J., Prapapan O., Komine M., Ohtsuki M. (2016). Role of matrix metalloproteinases in photoaging and photocarcinogenesis. Int. J. Mol. Sci..

[4] Fruntelată R.F., Bakri A., Stoica G.A., Mogoantă L., Ionovici N., Popescu G., Pîrşcoveanu D.F.V., Raicea A., Ciurea M.E. (2023). Assessment of tumoral and peritumoral inflammatory reaction in cutaneous malignant melanomas. Rom. J. Morphol. Embryol..

[5] Davis L.E., Shalin S.C., Tackett A.J. (2019). Current state of melanoma diagnosis and treatment. Cancer Biol. Ther..

[6] Brînzea A., Nedelcu R.I., Ion D.A., Turcu G., Antohe M., Hodorogea A., Călinescu A., Pirici D., Popescu R., Popescu C.M. (2019). Matrix metalloproteinases expression in lentigo maligna/lentigo maligna melanoma—A review of the literature and personal experience. Rom. J. Morphol. Embryol..

[7] Zob D.L., Augustin I., Caba L., Panzaru M.C., Popa S., Popa A.D., Florea L., Gorduza E.V. (2022). Genomics and epigenomics in the molecular biology of melanoma—A prerequisite for biomarkers studies. Int. J. Mol. Sci..

[8] Tas F. (2012). Metastatic behavior in melanoma: Timing, pattern, survival, and influencing factors. J. Oncol..

[9] Hofmann U.B., Westphal J.R., Zendman A.J.W., Becker J.C., Ruiter D.J., van Muijen G.N.P. (2000). Expression and activation of matrix metalloproteinase-2 (MMP-2) and its co-localization with membrane type 1 matrix metalloproteinase (MT1-MMP) correlate with melanoma progression. J. Pathol..

[10] Naik P.P. (2021). Role of biomarkers in the integrated management of melanoma. Dis. Mark..

[11] Zbytek B., Carlson J.A., Granese J., Ross J., Mihm M.C., Slominski A. (2008). Current concepts of metastasis in melanoma. Expert. Rev. Dermatol..

[12] Ricciardi E., Giordani E., Ziccheddu G., Falcone I., Giacomini P., Fanciulli M., Russillo M., Cerro M., Ciliberto G., Morrone A. (2023). Metastatic Melanoma: Liquid Biopsy as a New Precision Medicine Approach. Int. J. Mol. Sci..

[13] Mahumud R.A., Shahjalal M. (2022). The Emerging Burden of Genetic Instability and Mutation in Melanoma: Role of Molecular Mechanisms. Cancers.

[14] Davey M.G., Miller N., McInerney N.M. (2021). A Review of Epidemiology and Cancer Biology of Malignant Melanoma. Cureus.

[15] Sheng Y., Yanping C., Tong L., Ning L., Yufeng L., Geyu L. (2020). Predicting the Risk of Melanoma Metastasis Using an Immune Risk Score in the Melanoma Cohort. Front. Bioeng. Biotechnol..

[16] Sundararajan S., Thida A.M., Yadlapati S., Koya S. (2022). Metastatic Melanoma. StatPearls.

[17] Keung E.Z., Gershenwald J.E. (2018). The eighth edition American Joint Committee on Cancer (AJCC) melanoma staging system: Implications for melanoma treatment and care. Expert. Rev. Anticancer Ther..

[18] Pathak S., Zito P.M. (2022). Clinical Guidelines for the Staging, Diagnosis, and Management of Cutaneous Malignant Melanoma. StatPearls.

[19] Savage P. (2007). Malignant melanoma (non-metastatic). BMJ Clin. Evid..

[20] Gerlini G., Tripo L., Sestini S., Brandani P., Giannotti V., Gattai R., Borgognoni L. (2018). Melanoma metastases occuring 40 years after primary melanoma. Acta Oncol..

[21] Mervic L. (2012). Time course and pattern of metastasis of cutaneous melanoma differ between men and women. PLoS ONE.

[22] Bastian A., Nichita L., Zurac S., Tavascio F. (2017). Matrix metalloproteinases in melanoma with and without regression. The Role of Matrix Metalloproteinase in Human Body Pathologies.

[23] Singvogel K., Schittek B. (2024). Dormancy of cutaneous melanoma. Cancer Cell Int..

[24] Tejera-Vaquerizo A., Nagore E., Meléndez J.J., López-Navarro N., Martorell-Calatayud A., Herrera-Acosta E., Traves V., Guillén C., Herrera-Ceballos E. (2012). Chronology of metastasis in cutaneous melanoma: Growth rate model. J. Investig. Dermatol..

[25] Waheed Roomi M., Efremov E., Niedzwiecki A., Rath M. (2019). Matrix metalloproteinases-9 as a promising target for anti-cancer vaccine: Inhibition of melanoma tumor growth in mice immunized with syngeneic MMP-9 peptides. WCRJ.

[26] Hofmann U.B., Westphal J.R., Van Muijen G.N., Ruiter D.J. (2000). Matrix metalloproteinases in human melanoma. J. Investig. Dermatol..

[27] Anchan A., Finlay G., Angel C.E., Hucklesby J.J.W., Graham S.E. (2022). Melanoma Mediated Disruption of Brain Endothelial Barrier Integrity Is Not Prevented by the Inhibition of Matrix Metalloproteinases and Proteases. Biosensors.

[28] Peng K., Zhang Y., Liu D., Chen J. (2023). MMP2 is a immunotherapy related biomarker and correlated with cancer-associated fibroblasts infiltrate in melanoma. Cancer Cell Int..

[29] Mastalier Manolescu B.S., Lazar A.M., Ţiplica G.S., Zurac S.A., Reboşapcă A., Andreescu B., Popp C.G. (2024). MMP1, MMP9, MMP11 and MMP13 in melanoma and its metastasis—Key points in understanding the mechanisms and celerity of tumor dissemination. Rom. J. Morphol. Embryol..

[30] Hadler-Osler E., Fadnes B., Stylte I., Uhlin-Hansen L., Winberg J.-O. (2011). Regulation of matrix metalloproteinase activity in health and disease. FEBS J..

[31] He J., Qin M., Chen Y., Hu Z., Xie F., Ye L., Hui T. (2020). Epigenetic regulation of matrix metalloproteinases in inflammatory diseases: A narrative review. Cell Biosci..

[32] Tatti O., Arjama M., Ranki A., Weiss S.J., Keski-Oja J., Lehti K. (2011). Membrane-type-3 matrix metalloproteinase (MT3-MMP) functions as a matrix composition-dependent effector of melanoma cell invasion. PLoS ONE.

[33] Laronha H., Carpinteiro I., Portugal J., Azul A., Polido M., Petrova K.T., Salema-Oom M., Caldeira J. (2020). Challenges in Matrix Metalloproteinases Inhibition. Biomolecules.

[34] Jabłońska-Trypuć A., Matejczyk M., Rosochacki S. (2016). Matrix metalloproteinases (MMPs), the main extracellular matrix (ECM) enzymes in collagen degradation, as a target for anticancer drugs. J. Enzyme Inhib. Med. Chem..

[35] Islam M.T., Jang N.H., Lee H.J. (2024). Natural Products as Regulators against Matrix Metalloproteinases for the Treatment of Cancer. Biomedicines.

[36] Serraino G.F., Jiritano F., Costa D., Ielapi N., Battaglia D., Bracale U.M., Mastroroberto P., Andreucci M., Serra R. (2023). Metalloproteinases in cardiac surgery: A systematic review. Biomolecules.

[37] Gonzalez-Avila G., Sommer B., Mendoza-Posada D.A., Ramos C., Garcia-Hernandez A.A., Falfan-Valencia R. (2019). Matrix metalloproteinases participation in the metastatic process and theirdiagnostic and therapeutic applications in cancer. Crit. Rev. Oncol. Hematol..

[38] Bassiouni W., Ali M.A.M., Schulz R. (2021). Multifunctional intracellular matrix metalloproteinases: Implications in disease. FEBS J..

[39] Roomi M.W., Kalinovsky T., Niedzwiecki A., Rath M. (2017). Modulation of MMP-2 and -9 secretion by cytokines, inducers and inhibitors in human melanoma A-2058 cells. Oncol. Rep..

[40] Mondal S., Adhikari N., Banerjee S., Amin S.A., Jha T. (2020). Matrix metalloproteinase-9 (MMP-9) and its inhibitors in cancer: A minireview. Eur. J. Med. Chem..

[41] Winer A., Adams S., Mignatti P. (2018). Matrix Metalloproteinase Inhibitors in Cancer Therapy: Turning Past Failures into Future Successes. Mol. Cancer Ther..

[42] Mustafa S., Koran S., AlOmair L. (2022). Insights into the role of matrix metalloproteinases in cancer and its various therapeutic aspects: A review. Front. Mol. Biosci..

[43] Quintero-Fabián S., Arreola R., Becerril-Villanueva E., Torres-Romero J.C., Arana-Argáez V., Lara-Riegos J., Ramírez-Camacho M.A., Alvarez-Sánchez M.E. (2019). Role of matrix metalloproteinases in angiogenesis and cancer. Front. Oncol..

[44] Redondo P., Lloret P., Idoate M., Inoges S. (2005). Expression and serum levels of MMP-2 and MMP-9 during human melanoma progression. Clin. Exp. Dermatol..

[45] Hofmann U.B., Eggert A.A.O., Blass K., Bröcker E.B., Becker J.C. (2003). Expression of matrix metalloproteinases in the microenvironment of spontaneous and experimental melanoma metastases reflects the requirements for tumor formation. Cancer Res..

[46] Desch A., Strozyk E.A., Bauer A.T., Huck V., Niemeyer V., Wieland T., Schneider S.W. (2012). Highly invasive melanoma cells activate the vascular endothelium via an MMP-2/integrin αvβ5-induced secretion of VEGF-A. Am. J. Pathol..

[47] Leight J.L., Tokuda E.Y., Jones C.E., Lin A.J., Anseth K.S. (2015). Multifunctional bioscaffolds for 3D culture of melanoma cells reveal increased MMP activity and migration with BRAF kinase inhibition. Proc. Natl. Acad. Sci. USA.

[48] Cotignola J., Reva B., Mitra N., Ishill N., Chuai S., Patel A., Shah S., Vanderbeek G., Coit D., Busam K. (2007). Matrix metalloproteinase-9 (MMP-9) polymorphisms in patients with cutaneous malignant melanoma. BMC Med. Genet..

[49] Moogk D., da Silva I.P., Ma M.W., Friedman E.B., de Miera E.V.-S., Darvishian F., Scanlon P., Perez-Garcia A., Pavlick A.C., Bhardvaj N. (2014). Melanoma expression of matrix metalloproteinase-23 is associated with blunted tumor immunity and poor responses to immunotherapy. J. Transl. Med..

[50] Chen Y., Chen Y., Huang L., Yu J. (2012). Evaluation of heparanase and matrix metalloproteinase-9 in patients with cutaneous malignant melanoma. J. Dermatol..

[51] Salemi R., Falzone L., Madonna G., Polesel J., Cinà D., Mallardo D., Ascierto P.A., Libra M., Candido S. (2018). MMP-9 as a Candidate Marker of Response to BRAF Inhibitors in Melanoma Patients with BRAFV600E Mutation Detected in Circulating-Free DNA. Front. Pharmacol..

[52] Zurac S., Neagu M., Constantin C., Cioplea M., Nedelcu R., Bastian A., Popp C., Nichita L., Andrei R., Tebeica T. (2016). Variations in the expression of TIMP1, TIMP2 and TIMP3 in cutaneous melanoma with regression and their possible function as prognostic predictors. Oncol. Lett..

[53] Wylie S., MacDonald I.C., Varghese H.J., Schmidt E.E., Morris V.L., Groom A.C., Chambers A.F. (1999). The matrix metalloproteinase inhibitor batimastat inhibits angiogenesis in liver metastases of B16F1 melanoma cells. Clin. Exp. Metastasis.

[54] de Almeida L.G.N., Thode H., Eslambolchi Y., Chopra S., Young D., Gill S., Devel L., Dufour A. (2022). Matrix Metalloproteinases: From Molecular Mechanisms to Physiology, Pathophysiology, and Pharmacology. Pharmacol. Rev..

[55] Belotti D., Paganoni P., Giavazzi R. (1999). MMP inhibitors: Experimental and clinical studies. Int. J. Biol. Mark..

[56] Sobirzhanovna B.N. (2024). New Aspects of Pathology and Norms of Matrix Metalloproteinases. Web Med. J. Med. Pract. Nurs..

[57] Cathcart J.M., Cao J. (2015). MMP Inhibitors: Past, present and future. Front. Biosci. (Landmark Ed.).

[58] Hidalgo M., Eckhardt S.G. (2001). Development of matrix metalloproteinase inhibitors in cancer therapy. J. Natl. Cancer Inst..

[59] Marusak C., Bayles I., Ma J., Gooyit M., Gao M., Chang M., Bedogni B. (2016). The thiirane-based selective MT1-MMP/MMP2 inhibitor ND-322 reduces melanoma tumor growth and delays metastatic dissemination. Pharmacol. Res..

[60] Blackburn J.S., Rhodes C.H., Coon C.I., Brinckerhoff C.E. (2007). RNA interference inhibition of matrix metalloproteinase-1 prevents melanoma metastasis by reducing tumor collagenase activity and angiogenesis. Cancer Res..

[61] Marusak C., Thakur V., Li Y., Freitas J.T., Zmina P.M., Thakur V.S., Chang M., Gao M., Tan J., Xiao M. (2020). Targeting Extracellular Matrix Remodeling Restores BRAF Inhibitor Sensitivity in BRAFi-resistant Melanoma. Clin. Cancer Res..

[62] Li F., Zhi J., Zhao R., Sun Y., Wen H., Cai H., Chen W., Jiang X., Bai R. (2024). Discovery of matrix metalloproteinase inhibitors as anti-skin photoaging agents. Eur. J. Med. Chem..

[63] Ågren M.S., Auf dem Keller U. (2020). Matrix Metalloproteinases: How Much Can They Do?. Int. J. Mol. Sci..

